# Elevated Temperature-Induced Epimicrobiome Shifts in an Invasive Seaweed *Gracilaria vermiculophylla*

**DOI:** 10.3390/microorganisms11030599

**Published:** 2023-02-27

**Authors:** Luisa Düsedau, Yifei Ren, Minglei Hou, Martin Wahl, Zi-Min Hu, Gaoge Wang, Florian Weinberger

**Affiliations:** 1 Marine Ecology Division, GEOMAR Helmholtz-Zentrum für Ozeanforschung Kiel, Düsternbrooker Weg 20, D-24105 Kiel, Germany; 2 Alfred Wegener Institute, Helmholtz Center for Polar and Marine Research, Am Handelshafen 12, 27570 Bremerhaven, Germany; 3 College of Marine Life Sciences, Ocean University of China, Qingdao 266003, China; 4 Institute of Evolution & Marine Biodiversity, Ocean University of China, Qingdao 266003, China; 5 Ocean School, Yantai University, Yantai 264005, China

**Keywords:** epibacterial communities, 16S rRNA gene amplicon sequencing, elevated temperature, *Gracilaria vermiculophylla*

## Abstract

Epibacterial communities on seaweeds are affected by several abiotic factors such as temperature and acidification. Due to global warming, surface seawater temperatures are expected to increase by 0.5–5 °C in the next century. However, how epibacterial communities associated with seaweeds will respond to global warming remains unknown. In this study, we investigated the response of epibacterial communities associated with the invasive *Gracilaria vermiculophylla* exposed to 3 °C above ambient temperature for 4 months using a benthocosm system in Kiel, Germany, and 16S rRNA gene amplicon sequencing. The results showed that elevated temperature affected the beta-diversity of the epibacterial communities. Some potential seaweed pathogens such as *Pseudoalteromonas*, *Vibrio*, *Thalassotalea*, and *Acinetobacter* were identified as indicator genera at the elevated temperature level. Thirteen core raw amplicon sequence variants in the elevated temperature group were the same as the populations distributed over a wide geographical range, indicating that these core ASVs may play an important role in the invasive *G. vermicullophylla*. Overall, this study not only contributes to a better understanding of how epibacterial communities associated with *G. vermiculophylla* may adapt to ocean warming, but also lays the foundation for further exploration of the interactions between *G. vermiculophylla* and its epimicrobiota.

## 1. Introduction

Epibacterial communities on seaweeds play an important role in the health, development, and disease of their algal hosts [1,2,3], depending on both the algal host itself (e.g., algal species, age) [3,4] and many abiotic factors (e.g., salinity, temperature, and carbon dioxide) [3,5,6,7]. To date, it has been documented that elevated temperatures can cause shifts in epibacterial communities in seaweeds [3,5,8]. For example, the relative abundance of Rhodobacteraceae on *Fucus vesiculosus* increased from 20% to 50% when temperature increased from 5 °C to 25 °C, and OTU (operational taxonomy unit) diversity was highest at 15 °C [5]. Epibacterial communities on *F. vesiculosus* forma *mytili* were significantly affected by 5 °C higher temperatures over an 11 week period in a mesocosm experiment. Elevated temperature significantly reduced the growth of *F. vesiculosus* forma *mytili* by 20%. Meanwhile, indicator OTUs were used to compare the species with higher relative abundance between the control and elevated temperature levels. Compared to the ambient temperature (control), the relative abundance of the indicator OTU *Octadecabacter antarcticus* of the *Rhodobacter*-aceae family decreased from 4.72‰ to 0.46‰ at elevated temperature. However, the relative abundance of an indicator OTU of the Gammaproteobacteria remarkably increased from week 4 to week 11 at elevated temperature, with a concomitant decrease at ambient temperature [8]. Mensch et al. [3] showed that the temperature effect on the epibacterial communities of *F. vesiculosus* was stronger in summer than in spring, while the number of the indicator OTUs at elevated temperature increased significantly from 12 to 38 during the summer period.

*Gracilaria vermiculophylla* is a red alga native to the northwest Pacific [7,9]. Over the past 100 years, it has successfully invaded many coastal habitats worldwide, including the eastern Pacific, western Atlantic, eastern Atlantic, and Mediterranean [7,10]. The invasion of *G. vermiculophylla* has had a severe negative impact on local coastal community structure, species richness, and ecosystem function [11]. This species has been listed as one of the most invasive seagrasses in Europe [11,12,13]. *G. vermiculophylla* can protect itself from facultative pathogens [14] depending on the associated microbiota, and the alga also has a core microbiome that has been detected in all Asian, European, and American populations studied so far [15]. The core microbiome may provide certain important functions, such as the production of vitamin B12 [16], which are valuable to the algal hosts. Similarly, the epibacterial communities of the invasive *G. vermiculophylla* was important for its successful invasion. Saha et al. [11] found that invasive *G. vermiculophylla* populations were significantly better defended than native populations when tested against the bacteria of the invaded area, suggesting that invasive *G. vermiculophylla* may rapidly adapt to the bacteria of the invaded areas. In addition, invasive algae were shown to be more capable than native algae of forming new functional symbiotic associations with microbiota in newly invaded habitats [11].

The richness, abundance, and composition of epibacterial communities in *G. vermiculophylla* have been shown to be influenced by abiotic factors such as salinity and time [7]. In particular, temperature is considered to be a very important environmental factor for seaweed growth. However, global sea surface temperatures are expected to increase by 0.5–2.5 °C or even 5 °C over the next century in the context of climate change [17,18]. Given the crucial ecological role of epibacterial communities for *G. vermiculophylla* and other seaweeds, it is important to know how increased seawater temperature translates into shifts in the epimicrobiome of these marine organisms.

In this study, we investigated the effects of elevated temperature on the dynamic changes of epibacterial communities on invasive *G. vermiculophylla* using a benthocosm system in Kiel, Germany, and 16S rRNA gene amplicon sequencing. We characterized the effect of elevated temperature on the dynamic composition and diversity of the epibacterial communities. We also tested whether there were core microbiota and indicator species. Understanding the dynamics of epibacteria on *G. vermiculophylla* will help us to explore potential mechanisms behind its invasion.

## 2. Materials and Methods

### 2.1. Collection of G. vermiculophylla Populations

Forty individuals of *G. vermiculophylla* were collected from Falkensteiner Strand (54°23′53.7″ N, 10°11′25.7″ E) at the Kiel Fjord on 22 May 2019. The samples were brought to the laboratory within 2 h using a cooler box. They were maintained in 5 L aquaria with aeration in a climate chamber for 2 days at 15 °C and a salinity of 14 psu (close to the salinity observed at the sampling site).

### 2.2. Experimental Design and Setup

The temperature experiment was conducted at the Kiel benthocosm facility in 2000 L mesocosms [18] from 24 May to 28 August 2019. Briefly, seawater was constantly supplied from the Kiel Fjord via a pipeline at a depth of 1 m with a flow rate of 1.3 tank volumes per 24 h. The fluctuations of seawater parameters (temperature, pH, and salinity) in the benthocosms were the same as in the Kiel Fjord. The seawater temperature in the tanks was monitored by internal sensors and could be controlled automatically by a heating and cooling system. Ten outdoor tanks were divided into two groups. The temperature of five tanks was the same as the ambient seawater temperature in the Kiel Fjord (normal temperature, Nt), while the temperature of the other five tanks was maintained at 3 °C above the ambient seawater temperature (higher temperature, Ht). Each replicate (net bag) contained material from only one individual of *G. vermiculophylla*. In general, most specimens collected at Falkensteiner Strand originally weighed 2–3 g. A 1 g piece of each individual was cut off, transferred to a net bag, and placed in the tank. Each net bag contained a 1 g piece from a different individual. Four net bags containing 1.0 g of *G. vermiculophylla* were placed in each tank. In order to maximize the exposure of *G. vermiculophylla* to sunlight, the nets containing *G. vermiculophylla* were fixed in the center of each tank just below the water surface. Five replicates (five tanks) were designed for both Nt and Ht groups.

### 2.3. Sampling of Epibacteria from G. vermiculophylla

For epibacterial sampling for 16S rRNA gene amplicon sequencing, one net bag was removed from each tank at each sampling time (28 May, 28 June, 28 July and 28 August 2019). Then, 1.0 g of *G. vermiculophylla* from the bag was transferred to a sterilized 50 mL Falcon tube filled with 15 mL of sterile seawater (SSW) and 15 sterilized glass beads. The Falcon tubes were then shaken on an oscillator for 5 min. The resulting bacterial cell suspension in the Falcon tube was transferred to a new sterilized 50 mL Falcon tube and centrifuged at 12,000 rpm (4 °C) for 10 min. The supernatant was removed, and the pellet was preserved by the addition of 10 mL of 100% anhydrous ethanol. Then, 40–45 cm^3^ of nitrogen was pumped into the tubes to remove oxygen. The tubes were transported on ice to Qingdao, China, and immediately stored at −20 °C until further processing.

### 2.4. Genomic DNA Extraction and 16S rRNA Gene Amplicon Sequencing

Genomic DNA was extracted using the HiPure Soil DNA Kits (Magen, Guangzhou, China) according to the manufacturer’s protocols. The 16S rDNA V3–V4 region of the ribosomal RNA gene was amplified by PCR (95 °C for 2 min, followed by 30 cycles at 95 °C for 1 min, 60 °C for 1 min, and 72 °C for 1 min, and a final extension at 72 °C for 7 min) using primers 341F: CCTACGGGNGGCWGCAG, 806R: GGACTACHVGGGTATCTAAT. PCR reactions were performed in a triplicate 50 μL mixture containing 10 μL of 5× Q5@ Reaction Buffer, 10 μL of 5× Q5@ High GC Enhancer, 1.5 μL of 2.5 mM dNTPs, 1.5 μL of each primer (10 μM), 0.2 μL of Q5@ High-Fidelity DNA Polymerase, and 50 ng of template DNA. Related PCR reagents were from New England Biolabs, USA. Amplicons were extracted from 2% agarose gels and purified using the AxyPrep DNA Gel Extraction Kit (Axygen Biosciences, Union City, CA, USA) according to the manufacturer’s instructions. Purified amplicons were pooled in equimolar and paired-end sequenced (PE250) on an Illumina NovaSeqTM 6000 Sequencing platform according to the standard protocols. The raw reads were uploaded to the NCBI Sequence Read Archive (SRA) database (Accession Number PRJNA909032).

### 2.5. Sequence Quality Filtering and Taxonomy Annotation

Quality control and clustering were carried out using the DADA2 R package (version 1.14) [19]. Raw reads were filtered and truncated by removing reads containing unknown nucleotides and removing primer sequences. Then, a dereplicated list of unique sequences and their abundances was generated by taking the average of the positional qualities of the component reads. After that, paired-end denoised reads were merged as raw amplicon sequence variants (ASVs) with a minimum overlap of 12 bp. Chimera sequences were identified and deleted using the UCHIME algorithm [20]. After chimera removal, the denoised, chimera-free ASV sequences and their abundances were compiled.

The representative ASV sequences were classified taxonomically by a naïve Bayesian model using the RDP classifier (version 2.2) [21] based on the SILVA database (version 138.1) [22] with the confidence threshold value of 0.8. Sequences classified as unknown and chloroplasts were removed prior to subsequent analyses. The raw reads were uploaded to the NCBI Sequence Read Archive (SRA) database (accession number PRJNA909032).

### 2.6. Statistical Analysis

To show the species composition at both temperature levels, the abundance statistics of each taxon were visualized using Krona (version 2.6) [22]. The stacked bar plot of community composition was visualized in R using the ggplot2 package (version 2.2.1) [23].

Alpha diversity indices were calculated in QIIME 2 [24]. ASV rarefaction curves and rank abundance curves were plotted in the R package ggplot2 package (version 2.2.1). Welch’s t-test and Kruskal–Wallis H-test, as implemented in the R project Vegan package (version 2.5.3), were used to compare alpha diversity indices between different groups for significant differences [25].

Beta diversity indices were used to assess the diversity of epibacterial communities. Principal coordinates analysis (PCoA) based on Bray–Curtis distances was generated using the R-project Vegan package (version 2.5.3). PERMANOVA (Adonis) was used on the distance matrix to test for differences between treatments.

Core epibacterial microbiota were defined as taxa that were consistently present in 100% of the samples (regardless of their relative abundance in the community). Venn analysis was performed using the R project Venn Diagram package (version 1.6.16) to identify unique and common species or ASVs [26]. Species comparison between groups was calculated using Welch’s t-test in Vegan package (version 2.5.3). Species comparison between normal and elevated temperature groups was calculated using the Kruskal–Wallis H-test.

The indicator species associated with elevated temperature was calculated using the indicator value (IndVal) method. It can be applied at different taxonomic levels [27]. IndVal calculations and cross-validation tests were performed using the labdsv package (version 2.0-1) in the R project [28]. Indicator species were selected at the genus level with *p* ≤ 0.05 and IndVal > 0.7 [29].

To infer the functional potential of epibacterial microbiomes, the Kyoto Encyclopedia of Genes and Genomes (KEGG) pathway analysis of ASVs was inferred using Tax4Fun (version 1.0) [30]. Analysis of functional differences between groups was calculated using Welch’s t-test and Kruskal–Wallis H-test in the Vegan package (version 2.5.3).

## 3. Results

### 3.1. Sequencing Summary of 16S rRNA Gene Amplicon Sequencing

**Figure 1:**
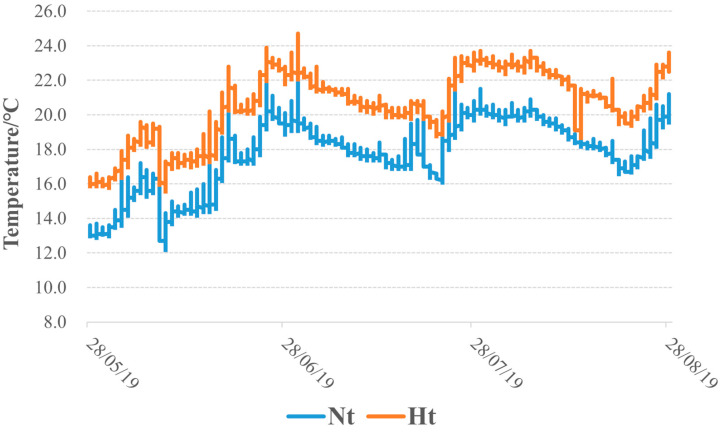
Temperature variation during the period from 28 May to 27 August 2019. Nt: normal seawater temperature. Ht: 3 °C higher temperature. The times on the X-axis represent the sampling dates.

The temperature changes during the experiment are shown in Figure 1. There were a total of 2,557,648 raw reads in the Nt group and 2,562,978 raw reads in the Ht group. After filtering, the numbers were 2,266,776 and 2,134,697, respectively. A total of 37,471 ASVs and 31,712 ASVs were obtained from the Nt and Ht samples, respectively. The ASV number of the Nt group was highest in June (Nt2, 12,711). The ASV number of the Ht group gradually decreased over time (Table S1). Good’s coverage indices together with the rarefaction curves indicated that sufficient sequencing was achieved to capture the full diversity of the epibacterial communities in this study (Figure S1).

### 3.2. Effects of Elevated Temperature on Epibacterial Diversity Communities

**Figure 2:**
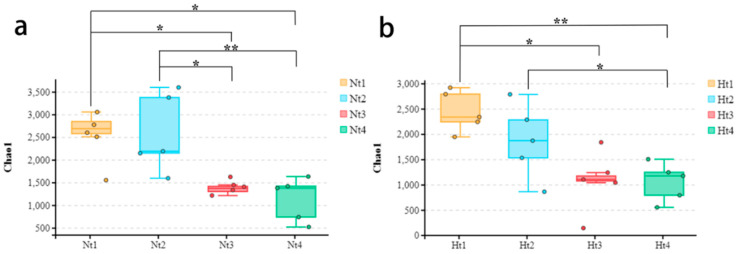
Chao1 diversity within both the Nt and Ht groups. (**a**) Chao 1 indices at normal seawater temperature. (**b**) Chao 1 indices at 3 °C higher temperature. Nt: normal seawater temperature. Ht: 3 °C higher temperature. 1, 2, 3, 4 represented samplings conducted in May, June, July and August, respectively. A single asterisk (*) indicated a significance level of *p* < 0.05, and a double asterisk (**) indicated a significance level of *p* < 0.01.

There were no significant differences in the Shannon and Chao 1 indices between the Nt and Ht groups. However, Chao1 showed significant differences within both the Nt and Ht groups (Figure 2a,b, Table S2; Kruskal–Wallis test, *p* ≤ 0.05). The Nt group significantly differed between May and July, between May and August, and between June and July. Chao 1 showed highly significant differences between June and August within the Nt group. In the Ht group, there were significant differences between May and July, and between June and August. For Chao 1, highly significant differences were found between May and August.

**Figure 3:**
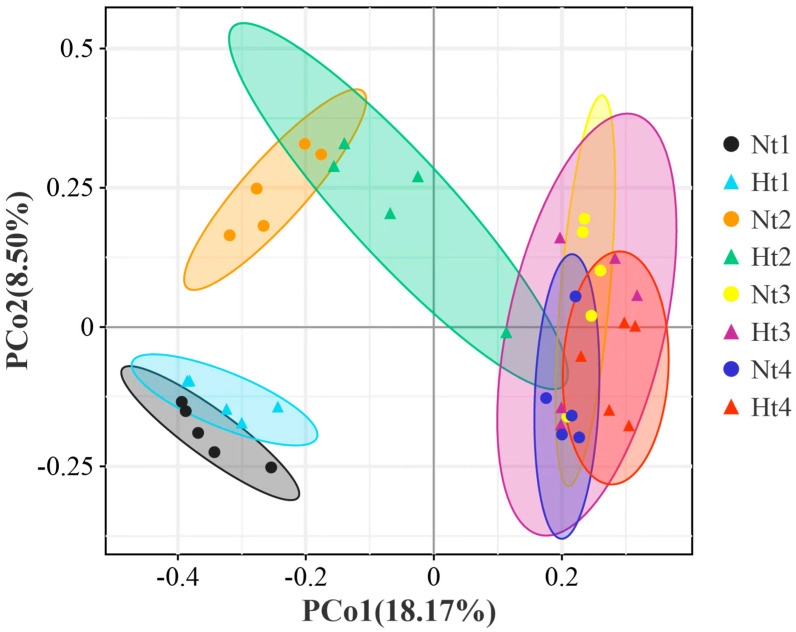
Beta diversity of *G. vermiculophylla*-associated epimicrobiota at two different temperatures. A principal coordinate analysis (PCoA) plot of *G. vermiculophylla* associated epimicrobiota based on Bray–Curtis distance. Different colors represented different months. Different dots indicate different samples. Nt: normal seawater temperature; Ht: 3 °C higher temperature; 1, 2, 3, and 4 represent May, June, July, and August, respectively.

Regarding beta diversity, a principal coordinate analysis (PCoA) plot based on Bray–Curtis distance revealed the changes in epibacterial communities at the two temperature levels. PCo1 and PCo2 explained 18.17% and 8.50% of the variation between samples, respectively (Figure 3). Except for July (Nt3 vs. Ht3; Adonis, *p* = 0.886), the bacterial community compositions in the other months were all significantly different for each of the Nt and Ht groups (Table 1). The difference in epibacterial community composition between the Nt and Ht groups was highly significant in August (Nt4 vs. Ht4; Adonis, *p* = 0.007). The results showed that Nt and Ht samples harbored different epibacterial communities.

### 3.3. Species Composition of Epibacterial Communities on G. vermiculophylla

**Figure 4:**
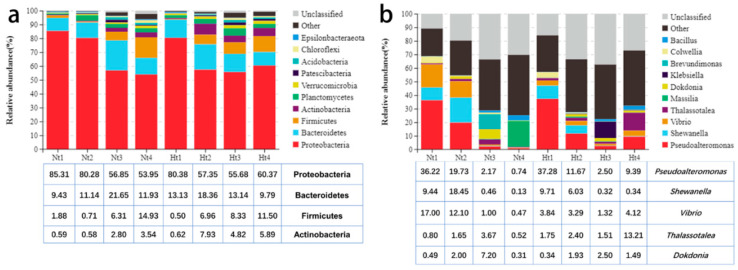
Epibacterial community composition of *G. vermiculophylla*. (**a**,**b**), The epibacterial distribution at phylum and genus level, respectively. Nt: normal seawater temperature; Ht: 3 °C higher temperature; 1, 2, 3, and 4 represent May, June, July, and August, respectively.

Proteobacteria were the most dominant phylum in all *G. vermiculophylla* samples regardless of heat treatment (Figure 4a). Proteobacteria, Bacteroidetes, Firmicutes, and Actinobacteria accounted for almost 90% of the species composition in the Nt group, of which the relative abundances were 69.10% ± 20.78%, 13.54% ± 7.09%, 6.00% ± 7.59%, and 1.90% ± 1.89%, respectively. The relative abundance of Proteobacteria in the Nt group gradually decreased over the months (Nt1, 85.31% ± 2.37% vs. Nt4, 53.95% ± 25.90%). As for the Ht group, the first four phyla were the same as in the Nt group. The relative abundances of Proteobacteria, Bacteroidetes, Firmicutes, and Actinobacteria were 63.45% ± 15.82%, 13.60% ± 5.46%, 6.82% ± 2.14%, and 4.81% ± 2.01%, respectively. It is worth noting that the relative abundance of Firmicutes in the Ht group increased over time, from 0.50% ± 0.20% in May to 11.50% ± 4.99% in August.

At the genus level, *Pseudoalteromonas*, *Vibrio*, *Shewanella*, and *Dokdonia* were the top four dominant genera with relative abundances of 14.72% ± 14.69%, 7.63% ± 8.16%, 7.12% ± 7.25%, and 2.49% ± 3.71%, respectively, in the Nt group (Figure 4b). The relative abundance of *Pseudoalteromonas* and *Vibrio* in the Nt group showed a decreasing trend with month. As for the Ht group, *Pseudoalteromonas*, *Thalassotalea*, *Shewanella*, and *Vibrio* were the dominant genera, for which the relative abundances were 15.21% ± 13.95%, 4.72% ± 6.70%, 4.10% ± 4.49%, and 3.14% ± 2.20%, respectively. The relative abundance of *Pseudoalteromonas* showed an initial decreasing trend from May to July, followed by an increasing trend from July to August.

### 3.4. Core Epibacterial ASVs on G. vermiculophylla at Two Temperature Levels

**Figure 5:**
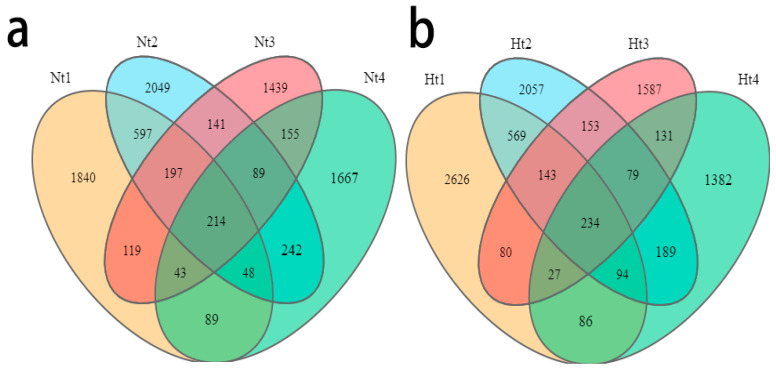
Core ASVs of different months. (**a**,**b**), Veen diagram showing the distribution of ASVs, displaying the extent of the core and exclusive ASVs across the 4 months in the Nt and Ht groups, respectively. Nt: normal seawater temperature; Ht: 3 °C higher temperature; 1, 2, 3, and 4 represent May, June, July, and August, respectively.

In this study, we defined core ASVs that were always present in 100% of the samples as the core epibacterial microbiota (regardless of their relative abundance in the community). In general, 214 core ASVs were detected in the Nt group (Figure 5a). In the Ht group, the number of core ASVs was 234 (Figure 5b). In the Nt group, these ASVs mainly belonged to Proteobacteria, Bacteroidetes, Firmicutes, and Actinobacteria with numbers of 145, 35, 14, and 7, respectively. The most abundant of these core bacteria were members of Caulobacterales (Caulobacteraceae, *Brevundimonas*, ASV000002), Vibrionales (Vibrionaceae, *Vibrio*, ASV000003), Alteromonadales (Pseudoalteromonadaceae, *Pseudoalteromonas*, ASV000001), and Alteromonadales (Shewanellaceae, *Shewanella*, ASV000005) (Table S1). As for the Ht group, the core ASVs also belonged mainly to Proteobacteria, Bacteroidetes, Firmicutes, and Actinobacteria. Their numbers were 156, 45, 15, and five, respectively. The most abundant of these core bacteria were members of Alteromonadales (Pseudoalteromonadaceae, *Pseudoalteromonas*, ASV000001), Alteromonadales (Colwelliaceae, *Thalassotalea*, ASV000006), Enterobacteriales (Enterobacteriaceae, *Klebsiella*, ASV000011), and Alteromonadales (Pseudoalteromonadaceae, *Pseudoalteromonas*, ASV000004). The relative abundance of the core ASVs in both groups varied over the different months.

### 3.5. Indicator Species over Time at Two Temperature Level

**Figure 6:**
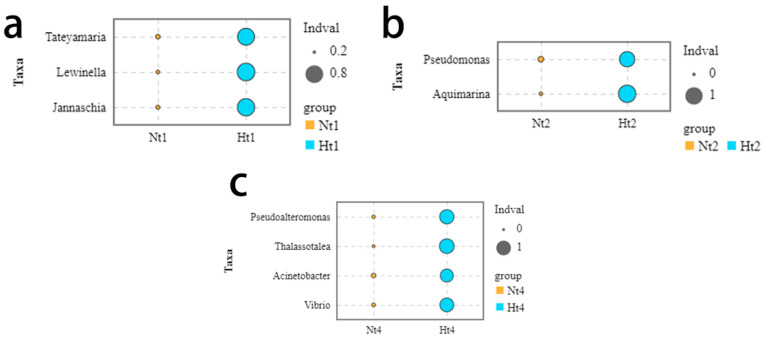
Extremely significant indicator genera at elevated temperature level. The horizontal and vertical axes represent different groups and indicator genera, respectively. The size of the bubbles in the figure represents the IndVal of the species between the Nt and Ht groups. Orange and blue represent the Nt group and the Ht group, respectively. Nt: normal seawater temperature; Ht: 3 °C higher temperature. (**a**–**c**) May, June, and August respectively. No highly significant indicator genera (*p* ≤ 0.05, Indval ≥0.7, relative abundance >0.3) were found in July in any of the groups.

The indicator value (Indval) method was used to test whether specific bacterial genera were associated with *G. vermiculophylla* at the two temperature levels. Taxa with *p* ≤0.05 and Indval ≥0.7 were considered as indicator genera [29]. In the Nt group, there were six indicator genera in May, 10 indicator genera in June, and two indicator genera in July, while no indicator genera were found in August (Table S2). As for the Ht group, there were 35 indicator genera in May, eight indicator genera in June, and 11 indicator genera in August, while no indicator genera were found in July (Table S2). We also selected some extremely significant indicator genera (*p* ≤ 0.05, Indval ≥0.7, relative abundance >0.3). Interestingly, no highly significant indicator genera were found in the Nt group at this level. In the Ht group, *Tateyamaria*, *Lewinella*, and *Jannaschia* were the extremely significant indicator genera in May (Figure 6a). *Aquimarina* and *Pseudomonas* were the highly significant indicator genera in June (Figure 6b). *Pseudoalteromonas*, *Thalassotalea*, *Acinetobacter*, and *Vibrio* were the highly significant indicator genera in August (Figure 6c).

### 3.6. Functional Prediction

**Figure 7:**
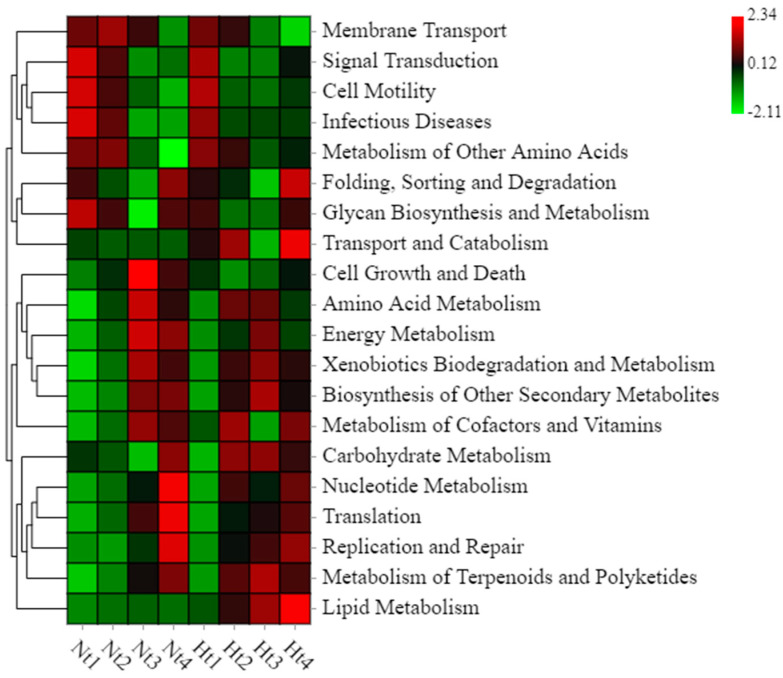
Functional prediction of epibacterial communities. KEGG pathway annotation of ASVs was inferred using Tax4Fun (version 1.0, Aßhauer et al., 2015 [30]). Nt: normal seawater temperature; Ht: 3 °C higher temperature; 1, 2, 3, and 4 represent May, June, July, and August, respectively. The red to green color scale represents the relative abundance of metabolic functions in the epimicrobiome.

Heatmaps were used to visualize the main differential functions (Figure 7). Compared to the Nt group, functions related to cofactor and vitamin metabolism, cell growth, and death were more abundant in the Ht group epimicrobiome in May (Welch’s *t*-test, *p* ≤ 0.05) (Figure S2). In June, the functions related to nucleotide metabolism and secondary metabolite biosynthesis were significantly higher in the epimicrobiome of the Ht group (Welch’s *t*-test, *p* ≤ 0.05). Functions related to infectious diseases were highly significantly enriched in the epimicrobial communities of the Ht group in August (Welch’s *t*-test, *p* ≤ 0.01).

## 4. Discussion

*Gracilaria vermiculophylla* has an optimum growth temperature of around 16 °C; therefore, artificial warming above this threshold will put pressure on its performance [31]. Performance will progressively decrease with increasing degree-days above this threshold [32] if acclimation does not result in a corresponding shift in the thermal optimum. In this study, the number of degree-days of supra-optimal thermal conditions for *G. vermiculophylla* was more than twice as high in the warmed treatment (399 vs. 126), and we suggest that the algae were consequently more stressed here than under ambient conditions. Stress can lead to a weakening of algal control over their bacterial epibionts [32].

In this study, we tested the influence of 3 °C higher temperature on the diversity and composition of the epibacterial communities of the invasive seaweed *G. vermiculophylla* using a benthocosm system from May to August 2019. Our results showed that a 3 °C increase in temperature could affect the beta diversity of *G. vermiculophylla*. However, there were almost no effects on alpha diversity during the 4 month benthocosm experiment. The dominant and indicator genera identified at the elevated temperature suggested that these genera may play an important role in response to the elevated temperature. In addition, the number of core ASVs was higher in the Ht group than in the control. The predicted KEGG metabolic pathways of the epibacterial communities changed with the shift of the epimicrobiota at the higher temperature level.

Increased temperature did not affect alpha diversity between the Nt and Ht groups. This is probably due to the ability of *G. vermiculophylla* to resist high-temperature stress [33,34,35]. The invasive *G. vermiculophylla* can tolerate relatively large temperature changes from 5 to 30 °C [33]. Hammann et al. [36] found that 60% of invasive *G. vermiculophylla* survived after being exposed to a temperature of 40.5 ± 0.5 °C for 3 h, whereas only 7% of native individuals survived such temperature stress. Invasive *G. vermiculophylla* populations showed significantly higher levels of HSP70 (heat-shock protein 70) expression. The strong resistance of the invasive *G. vermiculophylla* to heat stress may partly explain that there was no significant difference in alpha diversity between the Nt and Ht groups during the short period of 12 weeks.

Temperature is considered to be an important abiotic factor in shifting epibacterial communities of seagrasses [3,5,8,37]. Our results were generally consistent with this view, as higher temperature significantly structured the epibacterial communities of *G. vermiculophylla*. In this study, *Pseudoalteromonas*, *Vibrio*, *Thalassotalea*, and *Acinetobacter* were highly significant indicator genera for the Ht group in August based on *p* ≤ 0.05 and an Indval ≥0.7, as well as relative abundance >0.3 (Figure 6c). To date, the genus *Pseudoalteromonas* comprises more than 47 species [38]. Some species have been isolated from seaweeds and can grow at temperatures between 0 and 40 °C. Some members of the genus *Pseudoalteromonas* have been shown to have antibacterial activity, providing corals with defense against potential pathogens [39]. However, several studies have shown that *Pseudoalteromonas* are the opportunistic pathogens of algae [40,41,42,43]. *Vibrio* was the second indicator genus for the Ht group. Our results are consistent with the findings of Mensch et al. [8], as *Vibrio* was also the indicator genus on *F. vesiculosus* forma *mytili* in the 5 °C higher temperature group. *Vibrio* is widespread in marine environments around the world [44] and shows a strong temperature dependence; thus, its occurrence is more common in warm waters [45]. This may partly explain why *Vibrio* became the indicator genus in August when the temperature became relatively high (23 °C). In addition, *Vibrio* species were associated with coral diseases and were regulated by seawater temperature [45,46,47]. Therefore, the indicator species of *Vibrio* in this study may also be temperature-dependent. Most species of *Pseudoalteromona* and *Vibrio* have been identified as pathogens in seaweeds [40,41,42,48]. In August, the abundance of both *Pseudoalteromonas* and *Vibrio* was significantly higher in the Ht group than in the control, and there was no bleaching symptom in the Ht samples. Saha and Weinberger [14] found that potentially protective (Ralstonia sp., *Shewanella aquimarina*, *Tenacibaculum skagerrakense*, *Alteromonas stellipolaris*, *Tenacibaculum aestuarii*, *Cobetia marina*, *Nonlabens dokdonensis*, etc.) and pathogenic epibacteria (*Kordia algicida*, *Croceitalea eckloniae*, and *Pseudoalteromonas arctica*) were present on the surface of *G. vermiculophylla*. We speculate that there are protective bacterial strains that fight against pathogens and then prevent bleaching.

The genus *Thalassotalea* contains 19 species in the List of Prokaryotic Names with Standing in Nomenclature (LPSN) [49]. Bacteria of this genus have been isolated from seawater, marine sediments, and seaweeds [49,50,51]. The optimum temperature for most species of *Thalassotalea* is above 25 °C, while some species tolerate 37 °C [52,53]. This may indicate that *Thalassotalea* on *G. vermiculophylla* are able to grow at elevated temperatures. Some members of *Thalassotalea* can degrade polysaccharides derived from macroalgae [54], suggesting that *Thalassotalea* are potentially opportunistic bacteria. With regard to *Acinetobacter*, it has so far been identified as a human pathogen. Recently, some plant and human pathogenic bacteria have been identified in cultivated *Saccharina japonica* (personal communication of Prof. Gaoge Wang). Therefore, *Acinetobacter* may also be the opportunistic pathogenic bacteria for *G. vermiculophylla*. As there is relatively less information on the functions of *Thalassotalea* and *Acinetobacter*, the role of these indicator genera during heat stress needs to be further investigated. According to our indicator species, elevated temperature may increase the relative abundance of some potentially pathogenic bacteria on *G. vermiculophylla*.

Proteobacteria and Bacteroidetes were the dominant phyla for both Nt and Ht groups, which is consistent with other studies [37,55,56]. This also supported the view of the presence of generalist epibacteria common to many macroalgae [2]. It is interesting to note that the relative abundance of Firmicutes in the Ht group increased over time, from 0.50% in May to 11.50% in August. It has been reported that the predominance of Firmicutes can resist the effects of multiple stress parameters and may possess highly efficient energy production systems [57]. Therefore, the increase in Firmicutes may help *vermiculophylla* to survive better under high-temperature stress.

In this study, we identified the core epibacterial microbiota (present in 100% of the samples, regardless of their relative abundance in the community). Bonthond et al. [15] found 141 core OTUs in the epiphytic composition of *A. vermiculophyllum* (the synonym of *G. vermiculophylla*). After blasting the sequence data between Bonthond et al.’s core taxa and ours, 113 ASVs of sequences matched with 100% identity (Table S3). In total, 14 epiphytic ASVs in our results were also found in Bonthond’s study. Of these, the number of epiphytic ASVs in the control group was 10, while the number in the Ht group was 13. In Bonthond’s study, the *A. vermiculophyllum* samples came from the global scale, whereas in this study, the *A. vermiculophyllum* was collected from Kiel, Germany. This suggests that microbial community composition is more dependent on host conditions than on local and regional environments [58]. However, the role of core taxa in invasive seaweeds is crucial; the ability of an invasive seaweed to maintain specific taxa with beneficial functions may provide an advantage over competitors and perhaps even protection against detrimental microbiota from the environment that are unable to colonize the already populated niche [15].

Metabolic functions associated with Nt and Ht group epibacteria were predicted by Tax4Fun. Our results showed that the metabolic functions were altered by higher temperature. Compared with the Nt group, the functions related to the metabolism of cofactors and vitamins, the function related to the biosynthesis of secondary metabolites, and the function of infectious diseases were significantly higher in the epimicrobiome of the Ht group in different months. Functions related to the metabolism of cofactors and vitamins were more abundant in the epimicrobiome of the Ht group in May. Enzyme cofactors are known to be essential for the metabolism of microbial communities [59], while vitamins such as vitamin B12 are valuable for the algal hosts [16]. Functions related to the biosynthesis of secondary metabolites were significantly higher in the epimicrobiome of the Ht group in June. Macroalgae, especially red algae, are important sources of secondary metabolites such as halogenated aromatics [60]. Secondary metabolites such as bis-bromophenol (BDDE) ether have been isolated from the red alga *Polyopes lancifolius* [54]. Liu et al. [61] showed that BDDE has antifungal activity against several phytopatogenic fungi. In addition, the function of infectious diseases was significantly higher in the epimicrobiome of the Ht group in August. Lu et al. [62] found that the metabolic function of infectious diseases was associated with white feces syndrome in shrimp, and the increase in this function might lead to promote the invasiveness of other opportunistic pathogens. As this function was enriched in the Ht group, we speculate that an increase in temperature might affect the health status of the invasive *G. vermiculophylla*.

## 5. Conclusions

The epibacterial communities of *G. vermiculophylla* are important for its survival and invasion success. Our results suggest that temperature warming can significantly affect the beta-diversity and metabolic functions of the epibacterial communities of *G. vermiculophylla*. Indicator genera associated with elevated temperature suggest that warming supports the growth of potentially pathogenic bacteria. A recent study [63] showed that some adaptive changes in gene expression regulated by microRNA occurred after *G. vermiculophylla* invaded new areas in the European and North American coasts, allowing us to hypothesize that epibacteria are likely involved in such molecular interaction networks between the host *G. vermiculophylla* and associated microbiota. Future studies can focus on testing the epigenetic processes mediated by the epibacterial community structure of *G. vermiculophylla* at elevated temperatures.

## Figures and Tables

**Table 1:** Adonis test value of PCoA analysis.

Diffs	Df	SumsOfSqs	MeanSqs	F Value	R^2^	*p*-Value	Significant
Nt1 vs. Ht1	1	0.3561	0.3561	2.6577	0.2494	0.031	*
Nt2 vs. Ht2	1	0.4314	0.4314	2.0887	0.2070	0.011	*
Nt3 vs. Ht3	1	0.3312	0.3312	0.8277	0.0938	0.886	
Nt4 vs. Ht4	1	0.6326	0.6326	1.5713	0.1642	0.007	**
Nt1 vs. Nt2 vs. Nt3 vs. Nt4	3	2.8824	0.9608	3.4462	0.3925	0.001	**
Ht1 vs. Ht2 vs. Ht3 vs. Ht4	3	2.5944	0.8648	2.9528	0.3564	0.001	**

* *p* ≤ 0.05; ** *p* ≤ 0.01.

## Data Availability

The raw reads of this research were uploaded to the NCBI Sequence Read Archive (SRA) database (Accession Number PRJNA909032).

## Supplementary Materials

The following supporting information can be downloaded at https://www.mdpi.com/article/10.3390/microorganisms11030599/s1: Table S1. The ASV numbers of the Ht groups and Nt groups; Table S2. Numbers of tags and OTUs under temperature stress; Table S3. Number of alpha diversity indices of epibacterial communities of *A.vermiculophyllum*; Figure S1. Rarefaction curve for Good’s-coverage indices. The horizontal axis represents the number of tags sampled; Figure S2. Welch’s *t*-test of the mean for functional abundance.
