# Supplementary material for: Elevated Temperature-Induced Epimicrobiome Shifts in an Invasive Seaweed Gracilaria vermiculophylla
Source: Microorganisms. 2023 Feb 27;11(3):599. doi: 10.3390/microorganisms11030599 (PMC10058608; doi:10.3390/microorganisms11030599)
Supplement: Supplementary file 1 [file microorganisms-11-00599-s001.zip › microorganisms-2115279-supplementary.pdf]

Table S2. Number of Tags, OTUs under temperature stress.

Group ID	Total Tags	Taxon Tags	Singleton Tags	OTUs
**Nt1**	477427	465936	11491	12238
**Ht1**	515915	506380	9535	12008
**Nt2**	490000	477757	12243	12711
**Ht2**	575304	569430	5874	9199
**Nt3**	571861	568095	3766	6873
**Ht3**	572748	570347	2401	5296
**Nt4**	595409	591967	3442	5649
**Ht4**	602809	599818	2991	5209

Nt indicates that samples were taken from the normal temperature sea water. Ht indicates that samples were taken from the high temperature. Ht1, Ht2, Ht3 and Ht4 indicate that were taken from heated aquarium on different mouths (May, June, July and August).

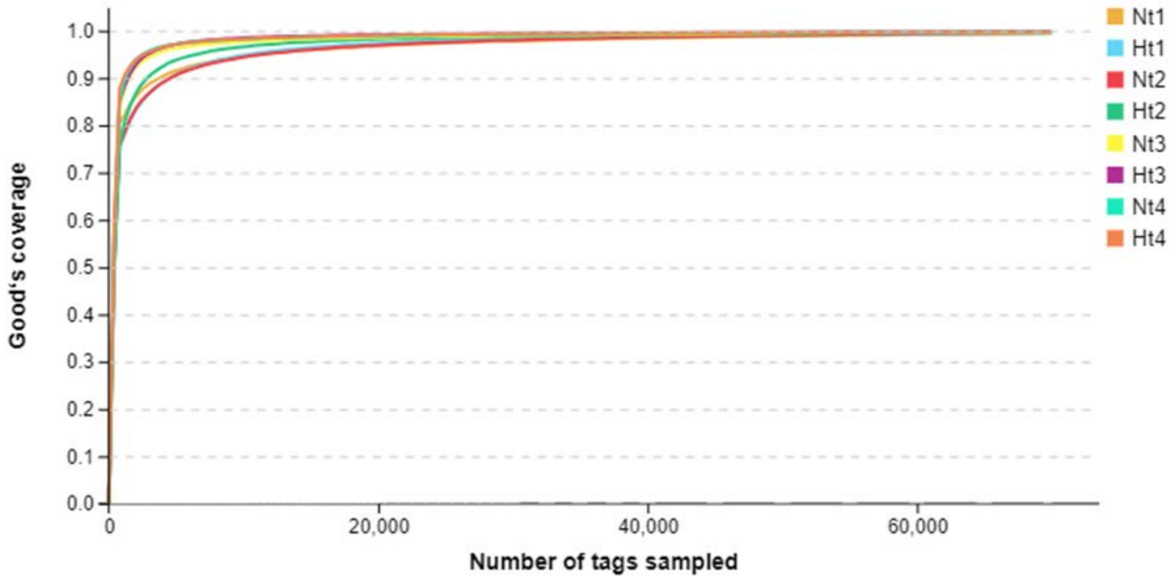

Figure S1. Rarefaction curve for Good's-coverage indices. The horizontal axis represents the number of tags sampled.

Supplementary Table S3. Number of alpha diversity indices of epibacterial communities of *A.vermiculophyllum*.

Group	Sobs	Shannon	Simpson	Chao1	Goods_coverage	Pielou
**Nt1**	2346.2	7.25511	0.9662	2393.88	0.99735	0.64993
**Nt2**	2375.4	8.02381	0.98524	2411.15	0.99766	0.71945
**Nt3**	1291.2	7.18793	0.9313	1318.07	0.99877	0.69593
**Nt4**	1069.8	6.90942	0.93364	1076.59	0.99955	0.69416
**Ht1**	2315.8	7.59126	0.96481	2357.57	0.99811	0.67989
**Ht2**	1741.6	8.18646	0.97768	1766.41	0.99865	0.77002
**Ht3**	985	7.16919	0.95807	997.699	0.99937	0.73927
**Ht4**	1016.2	7.19581	0.97531	1027.46	0.99945	0.7265

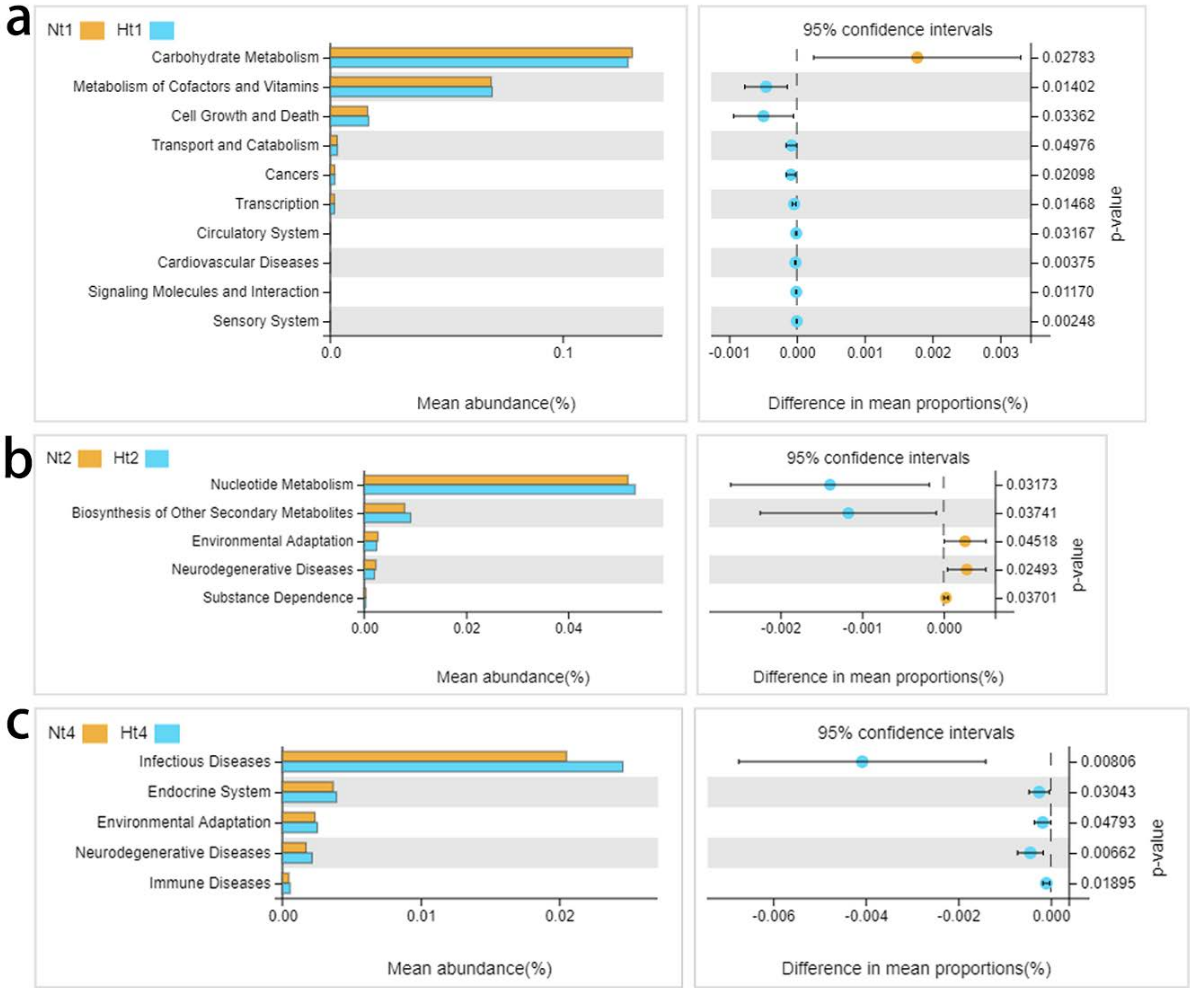

Figure S2. Welch's t test of the mean for functional abundance.
